# Molecular relationship between field and vaccine strain of measles virus and its persistence in Pakistan

**DOI:** 10.1186/1479-0556-10-1

**Published:** 2012-01-30

**Authors:** Masaud Shah, Sulaiman Shams, Ziaur Rahman

**Affiliations:** 1 Center of Excellence in Molecular Biology (CEMB), University of the Punjab, Lahore, Pakistan

**Keywords:** Measles, EPI, Outbreaks, Immunization, vaccines, Pakistan

## Abstract

**Background:**

Countrywide 5.9 million, 0-11 Month old children are immunized annually by EPI (Expended Program on Immunization) against 8 vaccine preventable diseases including measles and so on. Unfortunately the basic immunity centers are not uniform throughout the country. Each center provides services to about 27000 people which is inadequate. The purpose of this study was to explore the development of EPI Pakistan in terms of immunization of measles.

**Methods:**

Nucleotide sequences were analyzed by neighbor joining method (bootstrap test) using Bio- edit and MEGA-5 software to find evolutionary relationship between wild type measles strain and vaccine strain (Edmonston strain) used in Pakistan. For statistical analysis of data SPSS 16 was used.

**Results:**

Currently 1.3 vaccinators are working at each U C (union council) which according to national EPI policy should be at least 2. About 56% and 44% children of age 0-11 months did not received second dose of measles in the last two years respectively. Out of these 4231 cases which were reported last year, 1370 have received their first dose of measles vaccine.

**Conclusion:**

Seroconversion and seroprevalence study of the vaccine and field strain of measles virus is needed to confirm whether its failure is due to service unavailability or vaccine in-affectivity.

## Introduction

Immunization is a sole component of preventive medicine and is an important need of the day. Immunization reduces the cost of treating diseases and thus helps in poverty reduction and social and economic development of the country [1]. Globally EPI was initiated by the WHO In 1974 [2] and in Pakistan it was started in 1978 with the definitive objective of eliminating six common diseases (Tetanus, Diphtheria, Tuberculosis, Pertussis, Polio, and Measles) in the country which are vaccine preventable [3].

For many years childhood immunization program coverage remains low in Africa and Asia due to several reasons. These countries carry an inconsistent burden of global measles deaths. Approximately 610,000 infants and young children died in 2002 in these continents. In 1997 a new resolution was adopted by Eastern Mediterranean Region of the WHO to eliminate measles by 2010 [4]. The plan of National Immunization Days has remarkable impact on immunization coverage [5]. Annually 5.9 million 0-11 month old children are immunized by EPI Pakistan to protect them against 8 vaccine preventable diseases including measles. Unfortunately the basic immunization providing centers are not uniform in the country. About 6,000 fixed centers providing immunization services are present throughout the country. Each center provides services to about 27,000 populations, which is inadequate and its distribution is also not uniform. Presently 1.3 vaccinator are working at each UC (union council) which according to national EPI policy should be at least 2. In last 15 years the < 5 years mortality rates have shown some reduction but still it is 94 out of 1000 live births which is obviously terrifyingly [6]. About 56% and 44% children of age 0-11 months did not received measles II in the last two years respectively. Pakistan has made significant improvement in EPI coverage in comparison to India and Afghanistan. But more forceful implementation strategy is required to compete with other countries of the region.

## Material and methods

Several Government documents, survey reports and unpublished program documents were reviewed. Online searches were also made to find literature on coverage and surveillance of measles in Pakistan in websites of the World Health Organization (WHO), United Nations Children Fund (UNICEF) and other sources. EPI program official database was also analyzed for this study. 12 nucleotide sequences of polyprotein gene of measles virus strain reported in different areas of Pakistan and that of Edmonston strain used in measles vaccine in Pakistan were retrieved from NCBI gene Bank Data Base. To study evolutionary relation between wild type measles strain and Edmonston strain, nucleotide sequences were analyzed by neighbor joining method (bootstrap test) using Bio- edit and MEGA-5 software. For statistical analysis and graph construction SPSS 16 was used.

## Results

### 1. Need of Measles Vaccine

A highly infectious measles virus, have average of 12-18 cases spread from each index case in a fully susceptible population [7]. Measles virus behaves more like the smallpox virus in terms of transmission factors than the Polio virus and only replicates in humans. Measles virus is highly infectious, due to that reason a high level of population immunity is required to get herd immunity. The protection provided by maternal IgG decays by 6-9 months of age and infants becomes susceptible to measles infection. A vaccinated mother, who is never being exposed to circulating measles virus transfer less number of maternal IgG to her child as compare to mothers with a positive measles history. Natural measles infection tends to induce higher antibody levels than does measles vaccination. World Health Organization recommends vaccination at 9 months age which is significant for the reduction of mortality caused by measles [8]. Despite the relatively low (80-85%) seroconversion rates at 9 months of age most developing countries recommends vaccination of measles at this age because of high attack rates and serious disease among infants. To ensure optimum population immunity, all children should be given a second opportunity for measles immunization. Table 1 summarize Dose vise Schedule of different vaccine followed in Pakistan [9].

**Table 1:** Dose vise Schedule of different vaccine followed in Pakistan.

Vaccine	No. of Doses	Age
BCG	1	At birth
Trivalent OPV	4	At birth,6,10 and 14 weeks
Measles	2	At 9 month and 2^nd ^year of life
Pentavalent	3	At 6,10 and 14 weeks after birth

### 2. Vaccine Production in Pakistan

**Figure 1:**
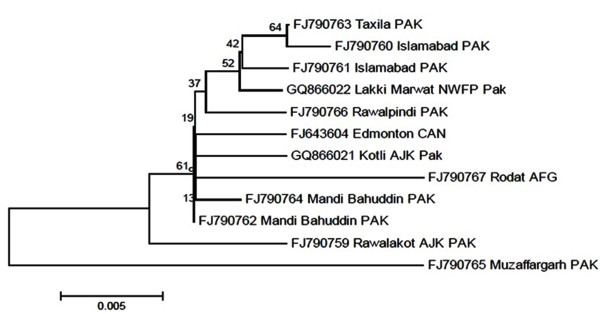
**Evolutionary Relationships of Taxa**. The percentage of replicate trees in which the associated Taxa clustered together in the bootstrap test (500 replicates) is shown next to the branches [25]. The tree is drawn to scale, with branch lengths in the same units as those of the evolutionary distances used to infer the phylogenetic tree. Abbreviations used: PAK-Pakistan, CAN-Canada and AFG-Afghanistan.

Killed measles vaccine was being used in the country after its licensing in 1963, but because of severe atypical pneumonia and high fever following subsequent exposure to measles vaccine it was stopped. A live attenuated measles vaccine originated from the Edmonston strain of measles virus isolated by Enders and Peebles in 1954 is now used in Pakistan since 1986. Measles vaccine has remained efficacious and does not appear to revert back in recipients because it is genetically very stable. The vaccine is produced by numerous passages of wild virus in various cell cultures to become attenuated. Although the Edmonston-derived vaccines have been developed in different types of cell cultures and have undergone different numbers of passages, nucleotide sequence analysis of selected genes shows minimal (< 0.6%) differences between these vaccines. Sequence analysis of nucleoprotein gene of both wild type measles virus and vaccine strain (Edmonston) used in Pakistan have a common ancestry. The evolutionary history was inferred using the Neighbor-Joining method [10]. The optimal tree with the sum of branch length = 0.07305901 is shown in Figure 1. The evolutionary distances were computed using the Maximum Composite Likelihood method [11] and are in the units of the number of base substitutions per site. The analysis involved 12 nucleotide sequences. All positions containing gaps and missing data were eliminated. There were a total of 456 positions in the final dataset. Evolutionary analyses were conducted in MEGA5 [12].

Current measles vaccine being used in Pakistan have been attenuated and produced in chick embryo fibroblasts. The minimum quantity of vaccine virus per human dose is determined by the national regulatory authority but is generally considered to be 1000 viral infective units [13]. The vaccine induces both humoral and cellular immune responses comparable to those following natural infection, although the serological titers are usually lower. IgM, IgG and IgA antibodies may be detected in both serum and nasal secretions, and IgG persists for many years. Declining antibody titer may be boosted by revaccination or by exposure to circulating measles virus.

### 3. Immunization Progress against Measles in Pakistan

**Figure 2:**
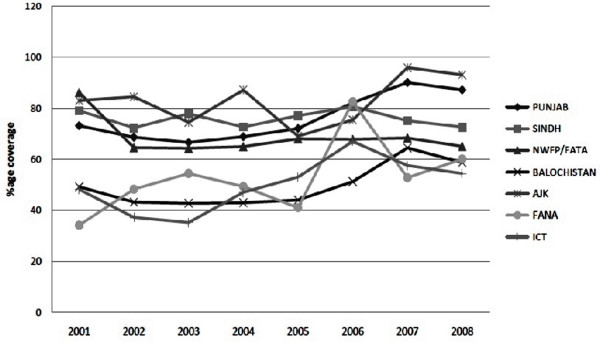
**Immunization of measles vaccine in Pakistan**.

The main body that has a key role in immunization of children and pregnant mothers is the extended program on immunization working at national institute of health Pakistan. The target of EPI is to immunize children of 0-11 months against eight EPI target. Annually about 5.9 million children and 6 million pregnant women are immunized against seven vaccine preventable diseases. It was predicted that measles mortality level will be reduced by 90% by 2010 as compared to 2000 but it has to be achieved still. The Recent advancement in vaccines and technologies has a considerable effect on immunization [14]. However these achievements are inadequate to reach the goal for polio eradication and measles elimination from the country. Coverage for measles was determined through different surveys conducted during the period of 2001-2010 (Figure 2). According to all surveys, the fully immunized child coverage ranged between 47% and 57% with an exception in the Pakistan Social and Living Standard Measurement Survey 2004-2005 [15], which reflected a higher achievement. The 2001 EPI survey shows that KPK (Khyber Pakhtunkhwa) province and FATA were the best performing regions with 86% immunization for measles. While the densely populated province "Punjab" shows 73% coverage.

**Figure 3:**
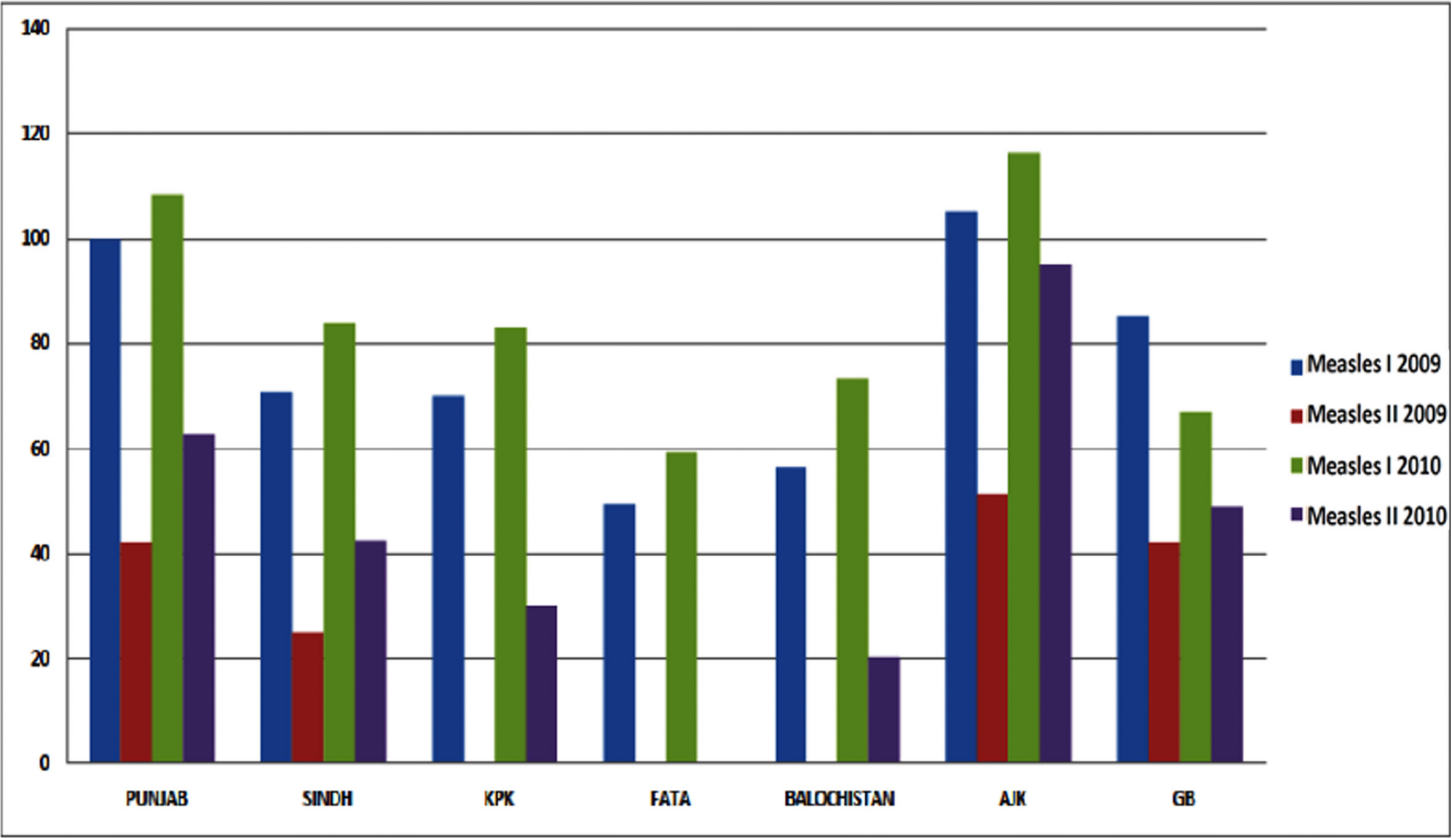
**2009-2010 survey results of Measles I and II immunization in different provinces of Pakistan**.

EPI Coverage Evaluation Survey 2006 [16] and Pakistan Demographic and Health Survey 2006-2007 [17] shows that only half of the target children were fully immunized with all antigens. In 2009 and 2010 EPI surveys indicate that in Punjab 100% children (0-11 month) received first dose of measles but only 42% and 62% children received the second dose in 2009 and 2010 respectively. Similarly 71% of 0-11 month children in Sindh, 70% in KPK, 49% FATA and 56% of children in Baluchistan received their first dose against measles in 2009 but all of them were deprived of their second dose for measles. In 2010, 43% of children in Sindh, 30% in KPK, 0% in FATA and only 20% of the children received the second dose for measles because of great devastation due to flood and terrorist activity in KPK and FATA regions (Figure 3). In 2011, 89% immunization has been achieved all over the country till last April [18].

### 4. Measles outbreaks in Pakistan

**Figure 4:**
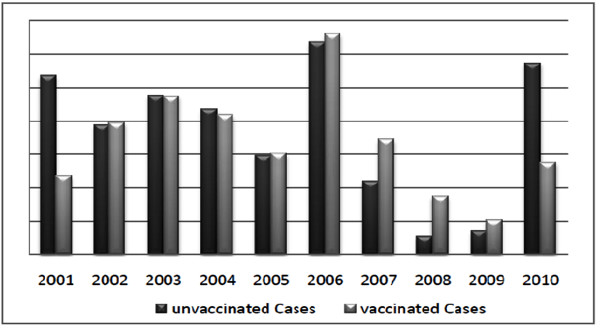
**Confirmed Measles cases reported in the last decade in Pakistan**.

Despite impressive progress in some parts of the world, measles still affects about 30 million persons each year, of which an estimated 610,000 die and many more suffer from complications and permanent sequelae. Despite of enormous efforts of EPI and other private sectors to eliminate measles, numbers of cases are reported each year. Not only non-vaccinated children but those which are previously vaccinated also develop the disease (Figure 4).

The main reason behind the question "that how the vaccinated children got the disease" is that most of them do not receive booster dose which is very important as recommended by WHO and Pakistan's regulatory authorities. In the last decade, years 2001, 2006 and 2010 years are considered to be epidemic. 3849 cases were reported in 2001 and 6480 in 2006 at EPI center Islamabad. Due to high efforts of bodies responsible for immunization, the number of reported cases reduced gradually in the next three years. However due to floods in 2010 and the terrorist activities in the past three years in KPK and FATA region a huge number of children failed to received their first dose of vaccine and almost all of them who have previously received their first dose, failed to boost up immunity against measles.

### 5. Reasons for poor coverage

EPI has raised its coverage up to 100% in some parts of the country and has got many successes, but still it have to tackle its goal of eliminating measles from the country. The main hurdles in its way to fight against measles are;

a) The key reason for this poor performance is the inadequate service delivery. Firstly the EPI centers are far away from the citizens and they cannot afford the cost to reach the center, secondly unavailability of vaccinators was found to be the main reasons. The 2006 Coverage Evaluation Survey of EPI indicated that 12.6% of mother's reasons for failing to immunize their children were distant vaccine center and unavailability of vaccinators [16].

b) The second most common contributing factor for low coverage is the Lack of recipient awareness about the immunization service and its benefits for their children. Low coverage in Punjab is the lack of parental awareness about the need for vaccination, as indicated by The Coverage Evaluation Survey Punjab 2003 [19].

c) Another main hurdle in vaccination progress is that, the health facility doctors neither refer the children for vaccination to the EPI center nor welcome any EPI activity at their health centers.

## Discussion

The administrative reports claimed high coverage but only around half of the targeted children were fully immunized as shown in all surveys conducted during 1995 to 2007 [15-17,19,20]. Concerns are found among different stake holders about the inconsistency between the reported data and independent assessments. Discrepancy in provincial performance was also evident in these surveys. Poor performance and limited access to the immunization service of EPI Pakistan, as revealed through a series of studies, is the most common cause for the large number of reported cases in the last decade [16,19,21-25]. Inadequate numbers of vaccinators was one of the main reasons for limited access to service [22,24]. All provinces have a much lower number of vaccinators than required according to the national policy except in Sindh (115%). Last year Proportions of vaccinators available against the standard were 52%, 70% and 72%, in Punjab, Khyber Pakhtunkhwa and Baluchistan provinces, respectively. A vaccinator working for 15-17 days every month making only 18-26 contacts each day is sufficient for an average-sized Union Council with a population of 25,000. However, this task becomes more challenging due to wide geographical dispersion of this target population. This inadequacy could be overcome by using EPI-trained lady health workers for delivering vaccination services. LHWs are embedded in and easily accepted by community. They have substantial potential for enhancing EPI coverage in their catchment area.

## Conclusion

Most of the children who have received their first dose against measles are often deprived of their second dose, due to which a large number of cases are reported each year. The low rates of coverage and dropout rates suggest that there is significant scope for improving efficiency of the EPI. Further, to confirm efficacy of measles vaccine we need seroconversion and seroprevalence study of the vaccine and field strain of measles virus in the country.

## Competing interests

The authors declare that they have no competing interests.

## Authors' contributions

MS and SS reviewed the literature, conducted all the statistical analysis and wrote the manuscript. ZR conceived the idea, guided MS and SS and edited the manuscript. All the authors read and approved the final manuscript.
